# In Vitro Models of Sturge–Weber Syndrome: Strengths, Limitations, and Future Goals

**DOI:** 10.3390/ijms27094100

**Published:** 2026-05-03

**Authors:** Ashleigh B. Manney, Christina L. Nemeth, Anne M. Comi

**Affiliations:** 1 Department of Neurodevelopmental Medicine, Kennedy Krieger Institute, Baltimore, MD 21205, USA; manney@kennedykrieger.org (A.B.M.); mertz@kennedykrieger.org (C.L.N.); 2 Hunter Nelson Sturge-Weber Syndrome Center, Kennedy Krieger Institute, Baltimore, MD 21205, USA; 3 Moser Center for Leukodystrophies, Kennedy Krieger Institute, Baltimore, MD 21205, USA; 4 Department of Neurology, Johns Hopkins School of Medicine, Baltimore, MD 21205, USA; 5 Department of Pediatrics, Johns Hopkins School of Medicine, Baltimore, MD 21205, USA

**Keywords:** Sturge-Weber syndrome, in vitro models, mosaic expression, GNAQ

## Abstract

Sturge–Weber Syndrome (SWS) is a rare congenital disorder presenting with a vascular malformation in the upper face and brain, causing impaired blood–brain barrier function and perfusion, increased calcium signaling, inflammation, and seizures. All these neuropathologic processes result in injury to the brain. The somatic GNAQ R183Q variant, which accounts for the majority of SWS cases, increases signaling through MAPK, PI3K, mTOR, and inflammatory pathways, primarily in endothelial cells. The discovery of this variant enabled the creation of transgenic and genetic animal and cell culture models. Generating in vitro models has been challenging due to the mosaic nature of SWS, and attempts to separate unaffected from mutant cells in primary culture have failed, limiting the utility of in vitro work. Ongoing in vitro work seeks to overcome these limitations, shape our understanding of SWS, and lead to translational advances in treatment and prevention by studying the affected molecular pathways and identifying future targets for therapy.

## 1. Introduction

Sturge–Weber Syndrome (SWS) is a congenital neurocutaneous and neurovascular disorder where blood vessels develop abnormally in the brain, skin, and eye, particularly affecting endothelial cells in the leptomeninges [1,2]. SWS is a rare disorder, affecting only 1 in 20,000 to 50,000 individuals [3]. Around 88% of SWS cases are caused by a somatic variant c.548G>A (p.R183Q) in the *GNAQ* gene that occurs early in fetal development, resulting in mosaic expression of the mutant protein in affected tissue. In the remaining percentage of SWS patients, other variants have been detected on a case-by-case basis, and many cases still have no known cause [3,4,5,6,7]. In mice, germline expression of the mutant protein (Gαq) was shown to be embryonic lethal [8,9,10]. In human affected tissue, usually including portions of affected skin or brain, the variant is present in endothelial cells to a much greater extent than other cell types [1]. Symptoms of SWS include epilepsy, migraine, venous stroke and stroke-like episodes, increased intraocular pressure and glaucoma, psychiatric disorders, growth hormone deficiencies, and sensory, motor, and cognitive deficits (Table 1). Symptoms due to SWS brain involvement tend to be progressive in infants and young children and stabilize as patients age. While most SWS patients present with a port-wine birthmark (PWB) on their head or neck, the presence of a PWB does not necessarily indicate that the patient has SWS. SWS brain involvement diagnosis is confirmed by neuroimaging by brain MRI with and without contrast, to determine whether the venous malformation involves the brain [2,3,11].

To better treat and prevent SWS progression, we must understand how the *GNAQ* variant gives rise to the affected phenotype. Understanding the molecular pathways involved is a necessary first step. Recent research efforts in mouse brain, human skin, and human brain samples have focused on pathways involved in blood–brain barrier (BBB) permeability and inflammation [12,13,14,15]. Compared to in vivo studies, in vitro work generally offers shorter timelines, more precise control over environmental conditions, reduced experimental variability, and minimal harm to living creatures in comparison to animal work. However, working with cells instead of entire organisms reduces the external validity of the results, and limiting variation in environmental conditions affects generalizability. While inbred strains and standardized housing conditions contribute to lower generalizability in animal models, working with cells outside an organism places them in conditions even further from human physiology. For example, endothelial cells rely on communication with other cell types and behave differently in vivo compared to isolated cultures, where neurons, astrocytes, and pericytes are not present. To circumvent this issue, established mutant endothelial cell cultures may be co-cultured with other cell types to investigate how communication between different cell types alters endothelial cell development and function. Cerebral organoid development incorporating the mutant endothelial cells is also a goal of the field.

Primary human tissue samples, which must come from the facial skin or the brain, are difficult to obtain. Additionally, the *GNAQ* variant follows a mosaic pattern of expression, making all primary cultures a mixture of unaffected wildtype (WT) and mutant cells. Assays performed on mixed cultures yield inconsistent results because mutant and WT cells proliferate at different rates, altering the culture composition over time [16]. To date, efforts to separate WT and GNAQ R183Q primary cells in a mixed tissue or culture sample remain unsuccessful. This review summarizes the molecular pathways involved in SWS, describes the current challenges in in vitro work, and lays a framework for addressing them.

## 2. GNAQ in Molecular Signaling Pathways

**Figure 1:**
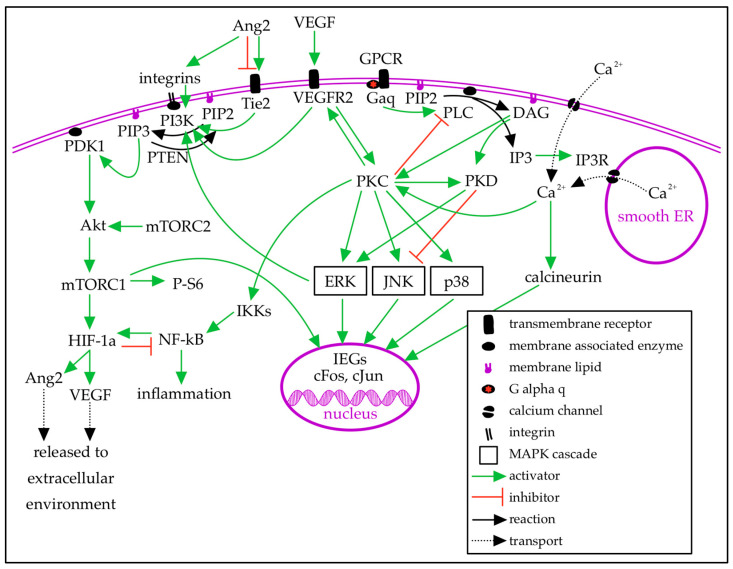
The *GNAQ* signaling pathway activates downstream MAPK, PI3K, mTOR, and inflammatory pathways. Gαq activates phospholipase C-β (PLC β) to hydrolyze the membrane lipid phosphatidylinositol 4,5-bisphosphate (PIP2) into inositol trisphosphate (IP3) and diacylglycerol (DAG). IP3 binds its receptors (IP3R) on the smooth endoplasmic reticulum (sER), triggering calcium release into the cytosol. Calcium release activates calcineurin, which alters gene expression. DAG activates protein kinase C (PKC) and protein kinase D (PKD), which influence mitogen-activated protein kinase (MAPK) pathways, ERK, JNK, and p38. MAPK pathway activation alters the expression of immediate early genes (IEGs), shaping cell behavior. PKC activates the IκB kinase complex (IKK), which degrades IκB proteins, releasing NF-κB for transcription of inflammatory factors. Phosphatidylinositol 3-kinase (PI3K), activated by ERK and cell surface receptors, phosphorylates PIP2 to phosphatidylinositol 3,4,5-trisphosphate (PIP3), reversed by phosphatase and tensin homolog (PTEN). PIP3 activates (PDK1), which phosphorylates Akt, increasing expression of mTOR complex 1 (mTORC1) and ribosomal phospho-S6 (P-S6). mTORC1 upregulates hypoxia-inducible factor 1α (HIF-1α), activating transcription of angiopoietin-2 (Ang2) and vascular endothelial growth factor (VEGF). VEGF and Ang2 are released into the extracellular environment and are primarily detected by endothelial cells. Ang2 is a partial agonist at the Tie2 receptor. Created in Notability v14.11.10 (https://notability.com/).

PWB and SWS are typically caused by a single-nucleotide polymorphism c.549G>A (p.R183Q) in the *GNAQ* gene that arises de novo during fetal development. It is hypothesized that the timing of the mutation during development determines the extent of tissue affected: the mutation may occur at variable time points, and the earlier the mutation occurs, the more tissue will be affected [12,17]. *GNAQ* encodes the G-protein alpha-q subunit (Gαq), which facilitates signal transduction between G-protein-coupled receptors (GPCRs) and intracellular signaling molecules (Figure 1). Gαq is coupled to many GPCRs, including muscarinic, adrenergic, vasopressin, endothelin, and angiotensin II receptors [18]. When a ligand binds to the extracellular receptor domain of a GPCR, the GPCR undergoes a conformational change, which signals the associated G-protein alpha subunit (Gα) to release GDP and bind cytosolic GTP. This reloads Gα: when GTP is bound, Gα is in its active state. Gα has intrinsic GTPase activity: once GTP is hydrolyzed to GDP, Gα becomes inactive (Figure 1). The R183Q variant changes an arginine residue to glutamine in the GTP-binding pocket of *GNAQ*, decreasing GTPase activity and prolonging Gαq activation, leading to overactivation of downstream effectors [3,11,19,20,21]. The Gαq subunit activates phospholipase C-β (PLCβ), which hydrolyzes phosphatidylinositol 4,5-bisphosphate (PIP2), a membrane lipid, to diacyl-glycerol (DAG) and inositol trisphosphate (IP3). IP3 is soluble in the cytosol and binds to IP3 receptors (IP3Rs) on the smooth endoplasmic reticulum (sER) to release intracellular calcium from the sER lumen [18,19,20]. Calcium release from the sER triggers influx of extracellular calcium through calcium-release-activated channels. Calcium activates calmodulin, which in turn phosphorylates calcineurin, leading to transcriptional changes [22,23]. DAG diffuses along the plasma membrane and primarily activates protein kinase C (PKC) [18,19,20]. This is the canonical view of the pathways impacted by the *GNAQ* R183Q change.

Mitogen-activated protein kinases (MAPKs) are a large family of protein kinases involved in intracellular signaling. Major subfamilies include ERK, which is involved in the regulation of cell growth and proliferation, and JNK and p38, which are involved in the cellular response to extracellular stressors [24,25,26]. PKC activates the MAPK/ERK, MAPK/JNK, and MAPK/p38 signaling pathways. Protein kinase D (PKD), activated by DAG and PKC, increases MAPK/ERK signaling and decreases MAPK/JNK signaling. ERK signaling is also increased by cytokines and growth factors [25,27,28]. The activation of PKC induces immediate early gene (IEG) expression via activation of MAPK signaling pathways. ERK, JNK, and p38 phosphorylate transcription factors to initiate IEG expression. IEGs, such as cFos and cJun, regulate gene expression in response to intracellular signaling events [29,30,31].

Changes to vascular endothelial growth factor (VEGF) signaling are hypothesized to play a role in SWS. VEGF promotes proliferation and permeability of vascular endothelial cells by its actions on Gαq-coupled receptors [32]. Disruption of VEGF signaling leads to the formation of vascular abnormalities [33,34]. VEGF expression is increased under conditions of hypoxia, mediated by hypoxia-inducible factor 1α (HIF-1α) and nuclear factor κB (NF-κB), and under conditions of oxidative stress, mediated by the PI3K/Akt/mTOR pathway [35,36]. Activation of GNAQ facilitates phosphorylation of VEGF receptor 2 (VEGFR2) via PLCβ and PKC [32]. Phosphorylated VEGFR2 activates the MAPK/ERK pathway by activating PKD via PKC (Figure 1). In vitro, PKD activation was blocked by VEGFR2 inhibition and decreased by PKC inhibition [28]. Greater VEGFR2 signaling leads to greater ERK activation and excess endothelial cell growth [33,37]. VEGFR2 also activates the PI3K/Akt/mTOR pathway [32,37].

Phosphatidylinositol 3-kinase (PI3K) is activated by MAPK/ERK and Tie2 signaling. PI3K phosphorylates phosphatidylinositol 4,5-bisphosphate (PIP2) to phosphatidylinositol 3,4,5-trisphosphate (PIP3). PIP3 is converted back to PIP2 by phosphatase and tensin homolog (PTEN), activated by the GPCR βγ subunit [23]. PIP3 activates 3-phosphoinositide-dependent kinase 1 (PDK1). PDK1 phosphorylates Akt, which leads to increased expression of mammalian target of rapamycin (mTOR; Figure 1) [38,39]. mTOR forms two protein complexes that regulate cell growth, proliferation, and survival in response to nutrients, hormones, and growth factors. mTOR complex 2 (mTORC2) directly activates Akt, which indirectly activates mTOR complex 1 (mTORC1) by removing inhibitors of mTORC1 [38,40]. The ribosomal protein phospho-S6 (P-S6) is a marker of mTOR activity [41]. mTOR upregulates a transcription factor that supports cell survival in hypoxic conditions [38,42]. Under normal conditions, HIF-1α is degraded. In hypoxic conditions, HIF-1α stabilizes and dimerizes, then binds to response elements to transcribe downstream genes [35]. HIF-1α reduces the activity of NF-κB. NF-κB is a family of transcription factors that induce transcription of inflammatory genes, including angiogenic and angiostatic factors, and stabilize HIF-1α [36,42]. Under baseline conditions, IκB proteins inhibit NF-κB. To induce inflammation, the IκB kinase complex (IKK) phosphorylates IκB, which is then degraded, releasing NF-κB to transcribe pro-inflammatory and apoptotic genes [42]. Activation of IKK via PKC leads to NF-κB activation and increased angiopoietin-2 (Ang2) expression [10,14].

Ang2 is a competitive antagonist to angiopoietin 1 (Ang1) at the tyrosine receptor kinase Tie2. In the presence of Tie1, Ang2 can act as a partial agonist at Tie2. Ang2 increases vascular permeability and angiogenesis in the presence of VEGF. Hypoxia and high blood glucose increase Ang2 expression [43]. Tie2 activates the MAPK/ERK, PI3K/Akt/mTOR, and NF-κB pathways [38,43]. Tie2 signaling is required to trigger NF-κB-mediated inflammation. Ang2 can also activate the PI3K/Akt/mTOR pathway independent of Tie2 via integrins (Figure 1) [14,43].

## 3. GNAQ Signaling Alterations in SWS

**Figure 2:**
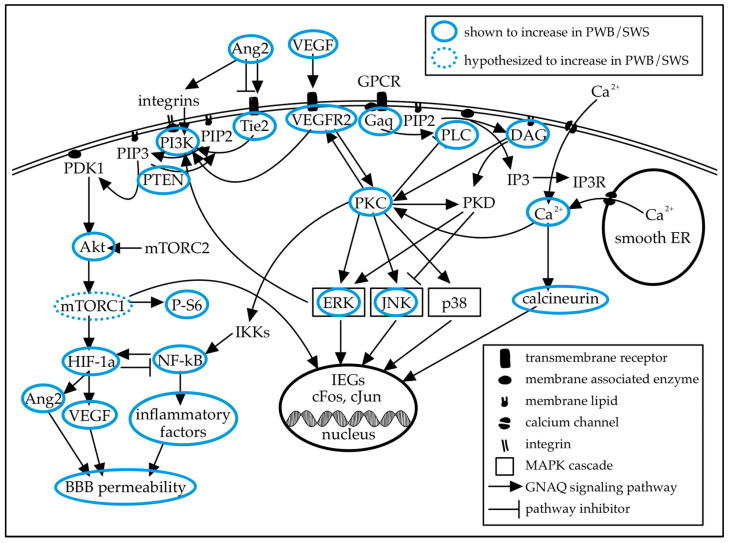
The GNAQ R183Q mutation present in SWS and PWB decreases the intrinsic GTPase activity of Gαq, causing prolonged activation of Gαq. Increases in expression or activity of many signaling molecules downstream of Gαq have been measured in samples of skin or brain tissue from patients with PWB or SWS. Increases in mTOR have been shown indirectly via P-S6 elevation, and direct measurement of mTOR is a target in future work. Created in Notability v14.11.10 (https://notability.com/).

In skin and brain tissue samples from SWS patients with GNAQ R183Q, increases in PLCβ, DAG, PKC, JNK, ERK, PI3K, AKT, P-S6, and intracellular calcium have been reported (Figure 2) [25,44,45,46]. In the leptomeningeal vessels of fixed SWS brain tissue, a larger proportion of endothelial cells demonstrate phosphorylated ERK than in epilepsy controls, as measured by quantitative analysis of immunohistochemistry images [1]. The MAPK/ERK and PI3K/Akt/mTOR pathways are known to be involved in angiogenesis and vessel permeability. In PWB skin samples, JNK and ERK are overactivated early in postnatal development, followed by AKT and PI3K activation in early childhood. In later childhood, PLCβ and PKC are overactivated, along with continued PI3K overactivation [25,44]. In GNAQ R183Q immortalized human endothelial cells, constitutive Gαq signaling was shown to increase intracellular calcium release from the sER, which increases extracellular calcium influx. Elevated intracellular calcium activates calcineurin, leading to increased dephosphorylation of NFAT transcription factors, allowing them to enter the nucleus and activate transcription [22,23,47]. Consistent with studies on patient tissue, immortalized R183Q cells have shown increased phosphorylated ERK. In contrast to findings in patient tissue, immortalized cells showed increased PTEN activation and downstream decreases in Akt and P-S6 when cultured with VEGF. These differences were not present in other culture conditions [23]. Previously, short-term cultures from patient tissue showed increased Akt [11]. More work is needed to determine how culture conditions and immortalized cell lines impact the clinical relevance of in vitro models.

While mTOR has not been directly measured in SWS tissue, P-S6 expression in leptomeningeal vessels, which is increased in the brain tissue of SWS patients compared to epilepsy controls, indicates increased mTOR activity in the cells of SWS-affected leptomeningeal vessels (Figure 2). This study was conducted on fixed tissue, and genotyping for the *GNAQ* variant was not possible for these samples [46]. A recent clinical trial demonstrated that treatment with oral sirolimus, an mTOR inhibitor, improved cognitive function and quality of life in patients with SWS [48].

Overexpression of VEGF, VEGFR2, and Tie2 has been reported in SWS GNAQ R183Q human tissue samples (Figure 2) [49]. VEGF is known to be important in angiogenesis, and higher urine VEGF levels in SWS patients have been associated with better neurologic function and less frequent seizures [50]. However, VEGF signaling has not been thoroughly investigated in relation to SWS [28]. The vascular anomalies caused by VEGF disruption are catastrophic in early development, and less severe in adulthood, which parallels the manifestation of SWS over the lifespan. Disruptions to VEGF signaling are often embryonic lethal in mice. Knockout of VEGF or its receptors, deletion of one VEGF allele resulting in expression at half the normal level, and overexpression of VEGF at two to three times the normal level all resulted in nonviable embryos due to vascular defects [32,34,35,51]. This suggests a low tolerance for disruption of VEGF signaling. Additionally, rodent studies of hyperoxia have demonstrated that disturbances in VEGF signaling during development disrupt retinal angiogenesis, leading to retinopathy [33]. VEGF and Ang2 increase in expression in response to hypoxia, oxidative stress, and high blood glucose [35,36,43]. Increased expression of Ang2 has been observed in human endothelial cells and mouse brain tissue expressing human GNAQ R183Q [10,14]. Ang2 overexpression is embryonic lethal in mice, and has been shown to interfere with the formation of normal blood vessels and induce BBB breakdown [43,52]. Similar mechanisms may be in play when *GNAQ* c.548G>A is expressed in all cells [9].

HIF-1α is found to be upregulated in abnormal leptomeningeal blood vessels of SWS patients with the GNAQ R183Q variant; however, it is not upregulated in affected cortical vessels of those same patients. Leptomeningeal HIF-1α upregulation in GNAQ R183Q patients is not due to hypoxia from poor perfusion, as surrounding non-endothelial cells do not demonstrate increased HIF-1α. High expression of HIF-1α in endothelial cells promotes proliferation by increasing growth factor expression, including VEGF [49]. There is bidirectional regulation between HIF-1α and NF-κB. The GNAQ R183Q variant has also been shown to increase NF-κB expression in endothelial cells and to increase production of inflammatory factors downstream of NF-κB. In human endothelial colony-forming cells engineered to express GNAQ R183Q, blocking NF-κB with a proteasome inhibitor decreased Ang2 mRNA and protein levels [14], suggesting the increase in NF-κB due to elevated Gαq activity contributes to an increase in Ang2 in R183Q cells (Figure 2).

The GNAQ R183Q mutation increases BBB permeability: transgenic mice expressing *GNAQ* c.548G>A showed greater staining of the brain than littermate controls after injection with Evans blue dye, indicating greater vascular permeability. These mice also showed discontinuous claudin-5 expression, a tight junction protein important for BBB integrity [8,12]. Disruption of claudin-5 increased BBB permeability and exacerbated kainate-induced seizures in a mouse model of temporal lobe epilepsy [53].

Before the GNAQ R183Q gene variant was linked to SWS in 2013, studies of skin and brain tissue, and of primary cells cultured from individual patients, provided insight into the molecular underpinnings of SWS pathology. Fibroblasts were cultured from skin biopsies of four SWS patients, and fibronectin mRNA and protein levels were increased in samples from affected (PWS) skin compared to unaffected skin. Fibronectin mRNA and protein levels were also increased in surgical brain tissue from SWS patients, as compared to surgical epilepsy and postmortem controls [54]. Fibronectin expression was further analyzed by in situ hybridization in brain samples from SWS patients and epilepsy controls, and skin samples from affected and unaffected skin in SWS patients. In SWS cortical tissue, fibronectin mRNA levels were reduced in meningeal vessels and increased in parenchymal vessels. Fibronectin expression in blood vessels was unchanged in PWS skin samples [55]. Later, early-passage fibroblasts isolated from SWS skin biopsies showed increased levels of several proteins supporting cellular proliferation and decreased levels of several proteins involved in regulating cellular proliferation. Paired samples of skin biopsies from affected (PWB) and unaffected skin from four SWS patients were tested, and protein was recovered from early-passage fibroblasts. Bulk protein concentration was measured by the Lowry protein assay. Individual proteins were quantified by iTRAQ (isobaric tag for relative and absolute quantification), a method that quantifies protein concentrations across multiple samples simultaneously using isobaric tags in mass spectrometry [56,57,58].

## 4. Challenges to Modeling SWS In Vitro

Until 2013, the largest obstacle to modeling SWS was the unknown cause [11]. Discovery of the GNAQ R183Q variant responsible for 88% of SWS cases allowed for the generation of animal and cell models of SWS (3). The GNAQ R183Q variant is not present in all patients with SWS. Other variants, including GNAQ R183L, GNAQ R183G, GNB2 K78E, and multiple substitutions at the GNA11 R183 locus, have been reported in individual patients, and in some cases, the variant has not been identified [3,4,5,6,7].

Currently, the largest barrier to establishing a long-term primary cell model of SWS has come from the mosaic pattern of expression (Table 2). A challenge in interpreting human tissue samples has been the lack of a marker for the GNAQ R183Q variant within tissue sections. Endothelial cells have been cultured from primary tissue samples obtained from SWS patients. However, primary tissue samples from the affected skin of patients with the GNAQ R183Q variant contain a mixture of cells expressing WT or mutant GNAQ [11]. Primary cultures have shown the variant is expressed in 24–34% of endothelial cells in affected brain tissue, compared to 8–9% of other cell types [15]. No method has been successful for distinguishing or separating GNAQ R183Q cells from a mixed live culture. Single-cell in situ hybridization is a possible approach to determine the percentage of WT or mutant cells while preserving samples for further analysis [11]; to our knowledge, this approach has not been applied to SWS brain tissue samples. Single-cell cloning could be used to create genetically identical cultures, but sorting techniques can reduce cell viability. Working with only primary cultures would be more clinically relevant. If cultures were only kept short-term, this could limit interference from a changing ratio of WT to mutant cells, allowing work with mosaic tissue. However, human tissue samples are rare and difficult to obtain, and samples from different patients may differ in their cell-type ratios.

Various cell types have been transfected with the *GNAQ* variant (Table 3). The GNAQ R183Q variant was induced in HEK 293T cells by site-directed mutagenesis, along with a luciferase reporter plasmid. Quantification of luciferase activity demonstrated that GNAQ R183Q activated downstream effectors to a greater extent than controls and a lesser extent than GNAQ Q209L, an activating mutation common to uveal melanoma [11]. Endothelial colony-forming cells (ECFCs) derived from human umbilical cord blood were transduced with the GNAQ R183Q variant via lentiviral transduction. ECFCs are progenitor cells in circulating blood that give rise to proliferative vascular endothelial cells [59]. Induction of GNAQ R183Q increased PLCβ activity, measured by phosphorylation of PLCβ regulatory sites; treatment with a Gαq inhibitor returned PLCβ phosphorylation to baseline levels. RNA sequencing of GNAQ R183Q cells revealed increased expression of proteins involved in the PKC, NF-κB, and calcineurin signaling pathways, which are linked to inflammatory phenotypes [14]. Immortalized endothelial cells from human aorta were edited with CRISPR/Cas9 to contain GNAQ R183Q. Transendothelial electrical resistance, a measure of tight junction integrity and monolayer permeability [60], was reduced in edited cells, indicating greater barrier permeability. This phenotype was rescued by MEK inhibition in combination with Ang2 knockdown [61]. Human endothelial cells, made to express GNAQ R183Q by lentiviral transduction, were subjected to laminar shear stress at varying flow rates [62]. In vivo, endothelial cells are subject to mechanical forces from blood flow, which alter gene expression and intracellular signaling. Endothelial culture under simulated blood flow more closely approximates in vivo conditions of vascular development [63,64]. A characteristic of SWS-affected blood vessels is tortuous growth, disrupting laminar blood flow and slowing flow rates. GNAQ R183Q cells did not change in roundness or circumference when subjected to laminar flow, while control cells elongated and aligned with the direction of flow. RNA quantification by RT-qPCR demonstrated increased transcription of inflammatory and apoptotic genes in GNAQ R183Q cells in low flow conditions, as well as increased transcription of cell adhesion molecules under high flow [62]. Immortalized human dermal microvascular endothelial cells with GNAQ knockout by CRISPR/Cas9 were transfected with GNAQ R183Q. These cells showed impaired wound healing in a scratch wound assay, with decreased endothelial migration toward the wound and decreased angiogenic sprouting. Greater activation of calcineurin and downstream effectors was recorded in edited cells. Inhibition of calcineurin restored downstream signaling and partially rescued wound-healing and angiogenic sprouting [23].

## 5. In Vivo Models of SWS

Since 2013, multiple mouse models of the *GNAQ* c.548G>A variant have been created [8,9,10,14]. Animal models offer an alternative source of *GNAQ* c.548G>A cells, and models of global genetic mutation, rather than the mosaic genetic presence observed in human patients, may facilitate the development of in vitro models in which all cells in a culture express the same *GNAQ* allele.

A Cre-driven model has shown that greater than 50% expression of the *GNAQ* c.548G>A transcript is embryonic lethal, while expression at lower levels, such as localized mosaic expression, is not. For conditional expression of p.R183Q, mice were created with one copy of the *GNAQ* c.548G>A variant, with exons 4–7 of the *GNAQ* gene flanked by loxP sites. These mice were crossed with R26-CreERT2 mice to temporally control mutant protein expression via tamoxifen administration at P3. This was the first model to demonstrate inducible expression of the GNAQ R183Q variant [9]. Another recently developed Cre model found that endothelial-specific expression of GNAQ R183Q is sufficient for the growth of capillary malformations. This model used CRISPR/Cas9 gene editing to insert cDNA for the *GNAQ* c.548G>A variant into the R26 vector. Initially, mice with the edited R26 vector were crossed with Cdh5-Cre mice, resulting in embryonic lethality when the *GNAQ* c.548G>A variant was expressed in all endothelial cells. Further experiments used Cdh5-CreERT2 mice, with tamoxifen administration at P1 to induce transgene expression. Endothelial production of GNAQ R183Q from P1 resulted in the development of features observed in human capillary malformation, including vascular abnormalities, increased endothelial cell proliferation, and increased expression of endothelial cell-specific molecule 1 in endothelial tip cells [10,65]. The Comi lab has developed a mouse model of SWS in which all cells harbor the human *GNAQ* c.548G>A variant, and mutant expression is controlled by a Tet-On system under the *Tie2* promoter, induced by doxycycline administration. Staining with Evans blue dye showed increased BBB permeability in mice expressing GNAQ R183Q. Immunohistochemistry showed increased P-S6 expression, discontinuous claudin-5 staining, and increased microvessel length and diameter in mice expressing GNAQ R183Q [8].

Transgenic mice that contain the *GNAQ* c.548G>A variant in all their endothelial cells, as opposed to a scattered population of cells as seen in human patients, offer unique advantages for in vitro work. In theory, all cells isolated from these mouse models would contain the *GNAQ* c.548G>A variant, and incubation with the appropriate drug could activate transcription of *GNAQ* c.548G>A in all cells at once. This would generate homogenous cultures without the need to separate mixed cultures. Additionally, transgenic mouse models allow temporal control over induction of gene expression. In culture, expression could be induced in all cells at precise time points to monitor cell growth and signaling (Table 2).

## 6. Future Directions in Modeling SWS

Future work on SWS should prioritize establishing relevant cell lines of WT and GNAQ R183Q cells. Endothelial cells demonstrate the GNAQ R183Q variant to a greater extent than other cell types [1] in cell-sorting experiments using human SWS tissue. Hence, primary efforts should focus on generating endothelial cell lines. In monoculture experiments, changes in GPCR signaling and the expression of relevant genes and proteins can be measured to elucidate cell signaling mechanisms. Mixed with other cell types or incorporated into more complex cell culture systems, interactions of GNAQ R183Q cells with their environment in the BBB can further validate mechanisms, and such systems could be used to identify targets for therapy. Endothelial cells, along with astrocytes, pericytes, and neurons, make up the neurovascular unit. Interactions between these cells control BBB permeability and stability. Endothelial cells, particularly brain microvascular endothelial cells, are necessary to form tight junctions and limit molecular passage across the BBB [66]. Characterization of GNAQ R183Q endothelial cell interactions with other cells of the neurovascular unit would contribute to understanding the SWS phenotype and could identify therapeutic targets.

Prospective drugs can be tested in cells to screen for potential clinical utility, including, but not limited to, medications such as sirolimus, an mTOR inhibitor, and Epidiolex, an endocannabinoid, which have previously shown promising results in clinical trials [12,48,67,68,69]. Oral sirolimus, administered over 6 months, improved patient scores on the Pattern Comparison Processing Speed Test. Outcomes on grip strength and dexterity tests, the Patient-Reported Outcomes Measurement Information System, and various cognitive tests from the NIH Toolbox did not show significant change. Treatment with sirolimus was shown to improve the quality of life in 10 of 10 SWS patients [48]. Oral cannabidiol (Epidiolex) up to 20 mg/kg/day improved neuroscore in nine out of nine patients, and improved quality of life in seven out of nine patients. Improvements in neuropsychiatric outcomes and motor function were also measured [68]. GNAQ R183Q cell lines can be used to investigate the effects of existing medications on molecular signaling pathways, as well as metabolism and growth. Reliable outcome measures can then be used to screen potential medications on a large scale. With the development of an in vitro assay linked to relevant clinical outcomes, GNAQ R183Q cells can be screened against multiple compounds at multiple doses to assess pharmacological activity. Future treatments aimed at correcting the single-nucleotide polymorphism can also be investigated in vitro to demonstrate editing efficiency before testing in animal models.

Finally, cerebral organoids derived from GNAQ R183Q patient-derived iPSCs would likely enable in vitro observation of abnormalities in both brain development and angiogenesis in SWS. Compared with traditional cell culture models, organoids offer a closer approximation of physiological tissue and development, with a three-dimensional arrangement of cells and extracellular matrix. While incorporating vascular structures improves organoid function and lifespan, it also increases the complexity of the model system, typically incorporating new cell types from different tissues or even host organisms [70]. Multiple methods have been successful in generating vascularized organoids: the organoid can be implanted into a host animal, where host vessels invade and perfuse the organoid; cerebral and vascular organoids can be created separately and fused together; differentiation into endothelial-like cells can be induced in some cells of the organoid; or the organoid can be embedded with or co-cultured with mature endothelial cells [70,71]. Vascular organoids of capillary malformation have been generated from patient-derived iPSCs and were shown to have increased cell density and vascular branch lengths, preserving clinically relevant phenotypes of capillary malformation [72]. Although these organoids lacked the GNAQ c.548G>A variant, vascularized cerebral organoids are a promising tool for further exploring SWS pathophysiology.

## 7. Conclusions

GNAQ signals via the calcium cascade to multiple downstream pathways, including MAPK, PI3K, mTOR, and inflammatory pathways. Overactivation of *GNAQ* increases signaling through these pathways. In SWS, *GNAQ* overactivation in leptomeningeal endothelial cells interferes with angiogenesis and BBB function. While multiple animal models have been established, there is still a need for in vitro models to more directly examine the mechanisms at play. Generating a renewable source of genetically homogeneous endothelial cells is the next step to overcome the obstacles posed by mosaic expression and mixed culture. The Tet-On expression system is promising, offering precise control over gene expression in mice and in cultured cells derived from them. Additional work, and even novel and more accurate model systems, will be necessary to directly probe disease mechanisms, identify modifiable targets for treatment, and test therapies and therapeutic strategies.

## Figures and Tables

**Table 1:** Clinical characteristics of SWS caused by capillary malformation in the brain, skin, and eye.

Brain	Eye	Skin
Epilepsy Stroke and stroke-like episodes Cognitive deficits Hemiparesis Visual field deficits Cortical calcification	Increased intraocular pressure Glaucoma Visual impairments	Facial port-wine birthmark/capillary malformation

**Table 2:** Approaches to modeling SWS in vitro. Primary culture is the most translationally applicable model, but is complicated by the mosaic nature of SWS and the inability to maintain a consistent ratio of mutant to wildtype cells. Transfection with less than 100% success results in mosaic samples and a loss of mutant cells over time. Single-cell seeding generates homogeneous cultures, but reduces cell viability. Isolating cells from animal models of SWS that employ controlled expression systems may be a viable method to generate a cell model of SWS.

Model	Source	Pros	Cons
Primary Culture	Human	Greatest clinical and translational relevance.	Mosaic expression, no method to separate WT and mutant cells.
iPSC Transfection	Human or animal	All cells genetically identical. Control of differentiation to produce different cell types.	Transfection less than 100% would reintroduce mosaic culture.
Single Cell Seeding	Human or animal	All cells genetically identical.	Low viability, isolation may trigger stress/inflammation pathways in some cell types.
Controlled Expression Systems	Animal	All cells genetically identical. Precise temporal control of gene expression.	

**Table 3:** In vitro models of SWS. Only one study has focused on patient-derived primary brain endothelial cells, and primary cultures were not sustained long-term.

Source	Model	Findings
Shirley et al., 2013 [11]	HEK293T, site-directed mutagenesis	GNAQ R183Q (SWS) stimulates the SRE promoter, but to a lesser degree than GNAQ Q209L (uveal melanoma).
Huang et al., 2017 [15]	Patient-derived brain endothelial, selected for CD31, 10 days in culture	The percentage of cells containing *GNAQ* c.548G>A was higher in endothelial cells than in other brain cell types.
Huang et al., 2022 [14]	Human umbilical cord blood-derived endothelial colony-forming cells, lentiviral transduction	GNAQ R813Q led to overexpression of Gαq, constitutive activation of PLCβ, and overactivation of PKC, NF-κB, and calcineurin pathways.
Nasim et al., 2025 [61]	Immortalized human aortic endothelial cells, CRISPR/Cas9	The trans-endothelial electrical resistance assay showed increased permeability in GNAQ R183Q endothelial cells, rescued by Ang2 knockdown and MEK inhibition.
Nasim et al., 2025 [62]	Human endothelial cells, lentiviral transduction	Endothelial cells with GNAQ R183Q do not elongate or align under shear stress and express inflammatory markers in low flow conditions.
Xu et al., 2026 [23]	Immortalized human dermal microvascular endothelial cells, GNAQ knockout by CRISPR/Cas9, lentiviral induction of GNAQ R183Q	GNAQ R183Q impaired migration and angiogenesis, and increased activation of calcineurin.

## Data Availability

No new data were created or analyzed in this study.

## Abbreviations

The following abbreviations are used in this manuscript:

Ang1	Angiopoietin 1
Ang2	Angiopoietin 2
BBB	Blood–brain barrier
DAG	Diacylglycerol
ECFC	Endothelial colony-forming cell
Gαq	G-protein alpha-q subunit
GPCR	G-protein-coupled receptor
HIF-1α	Hypoxia-inducible factor 1α
IEG	Immediate early gene
IKK	IκB kinase complex
IP3	Inositol trisphosphate
IP3R	Inositol trisphosphate receptor
iTRAQ	Isobaric tag for relative and absolute quantification
MAPK	Mitogen-activated protein kinase
mTOR	Mammalian target of rapamycin
mTORC1	Mammalian target of rapamycin complex 1
mTORC2	Mammalian target of rapamycin complex 2
NF-κB	Nuclear factor kappa B
PDK1	3-phosphoinositide-dependent kinase 1
PI3K	Phosphatidylinositol 3-kinase
PIP2	Phosphatidylinositol 4,5-bisphosphate
PIP3	Phosphatidylinositol 3,4,5-trisphosphate
PKC	Protein kinase C
PKD	Protein kinase D
PLCβ	Phospholipase C-β
P-S6	Phospho-S6
PTEN	Phosphatase and tensin homolog
PWB	Port-wine birthmark
sER	Smooth endoplasmic reticulum
SWS	Sturge–Weber syndrome
VEGF	Vascular endothelial growth factor
VEGFR2	Vascular endothelial growth factor receptor 2
WT	Wild type
